# Attributable mortality of acute kidney injury among critically ill patients with sepsis: a multicenter, retrospective cohort study

**DOI:** 10.1186/s12882-024-03551-9

**Published:** 2024-04-08

**Authors:** Dong-Hui Wang, Jin-Chao Zhao, Xiu-Ming Xi, Yue Zheng, Wen-Xiong Li

**Affiliations:** 1 Department of Surgical Intensive Care Unit, Beijing Chao-yang Hospital, Capital Medical University, 8 Gongren Tiyuchang Nanlu, Chaoyang District, 100020 Beijing, China; 2 Department of Clinical Laboratory, Xiangyang No.1 People’ s Hospital, Hubei University of Medicine, 441000 Xiangyang, China; 3 Department of Critical Care Medicine, Fuxing Hospital, Capital Medical University, Beijing, China

**Keywords:** Sepsis, Acute kidney injury, Attributable mortality, Propensity score matching

## Abstract

**Background:**

Sepsis and acute kidney injury (AKI) are common severe diseases in the intensive care unit (ICU). This study aimed to estimate the attributable mortality of AKI among critically ill patients with sepsis and to assess whether AKI was an independent risk factor for 30-day mortality.

**Methods:**

The information we used was derived from a multicenter prospective cohort study conducted in 18 Chinese ICUs, focusing on septic patients post ICU admission. The patients were categorized into two groups: those who developed AKI (AKI group) within seven days following a sepsis diagnosis and those who did not develop AKI (non-AKI group). Using propensity score matching (PSM), patients were matched 1:1 as AKI and non-AKI groups. We then calculated the mortality rate attributable to AKI in septic patients. Furthermore, a survival analysis was conducted comparing the matched AKI and non-AKI septic patients. The primary outcome of interest was the 30-day mortality rate following the diagnosis of sepsis.

**Results:**

Out of the 2175 eligible septic patients, 61.7% developed AKI. After the application of PSM, a total of 784 septic patients who developed AKI were matched in a 1:1 ratio with 784 septic patients who did not develop AKI. The overall 30-day attributable mortality of AKI was 6.6% (95% CI 2.3 ∼ 10.9%, *p* = 0.002). A subgroup analysis revealed that the 30-day attributable mortality rates for stage 1, stage 2, and stage 3 AKI were 0.6% (95% CI −5.9 ∼ 7.2%, *p* = 0.846), 4.7% (95% CI −3.1 ∼ 12.4%, *p* = 0.221) and 16.8% (95% CI 8.1 ∼ 25.2%, *p* < 0.001), respectively. Particularly noteworthy was that stage 3 AKI emerged as an independent risk factor for 30-day mortality, possessing an adjusted hazard ratio of 1.80 (95% CI 1.31 ∼ 2.47, *p* < 0.001).

**Conclusions:**

The overall 30-day attributable mortality of AKI among critically ill patients with sepsis was 6.6%. Stage 3 AKI had the most significant contribution to 30-day mortality, while stage 1 and stage 2 AKI did not increase excess mortality.

**Supplementary Information:**

The online version contains supplementary material available at 10.1186/s12882-024-03551-9.

## Background

Sepsis is defined as a life-threatening organ dysfunction caused by the host’s dysregulated response to infection [1], leading to one-third to one-sixth of global deaths [2–4]. Acute kidney injury (AKI) is characterized by a sharp decline in renal function caused by multiple causes in a short time. The harmful inflammatory cascade reaction of sepsis may be related to AKI [5, 6]. Sepsis is the leading cause of AKI in critically ill patients [7], and about 50% of septic patients are complicated with AKI [8, 9]. Compared to other causes of AKI, sepsis-related AKI leads to higher mortality [5].

Determining the precise proportion of sepsis-related deaths caused by AKI is a challenging task, particularly considering the intricate condition of septic patients in the intensive care unit (ICU). As such, accurate determination of the percentage of sepsis-related deaths that are specifically attributable to AKI remains elusive. Estimating AKI’s impact on mortality in critically ill septic patients is crucial for planning clinical trials [10]. Vaara ST estimated that the 90-day absolute excess mortality caused by AKI in ICU patients was 8.6% [10]. According to Jiang Yijia’s report, new-onset AKI was found to contribute to excess 30-day mortality of 8.1% in general ICU patients [11]. It is worth noting that studies on AKI-attributable mortality vary significantly among different studies. So, what exactly is the AKI excess mortality in ICU septic patients? Therefore, we undertook a comprehensive secondary data analysis of a large prospective multicentre cohort study. Employing propensity score matching (PSM) analysis, we meticulously reduced confounding biases to provide a more accurate estimation of the excess mortality specifically attributed to AKI. Additionally, we assessed whether AKI emerged as an independent risk factor for 30-day mortality among septic patients in the ICU.

## Materials and methods

### Study patients

The data for our study were derived from a prospective multicentre cohort study conducted by the China Critical Care Sepsis Trial (CCCST) working group. The study was carried out in 18 Chinese ICUs. The period was from 1 January 2014 to 31 August 2015. The CCCST study aimed to investigate the epidemiology and characteristics of critically ill patients with sepsis. Our study specifically targeted patients who met the sepsis 3.0 criteria [1] after ICU admission, thus being conclusively diagnosed with sepsis. The first day of diagnosis of sepsis was defined as the onset of sepsis. Following this diagnosis, patients were closely monitored for any signs of AKI that occurred within the first seven days. The exclusion criteria included: (1) patients already suffered from AKI of ICU admission; (2) AKI occurred before sepsis; (3) AKI occurred seven days later after the diagnosis of sepsis; (4) patients suffered chronic kidney disease (CKD), operated with nephrectomy or kidney transplantation; (5) patients received renal replacement therapy (RRT) for non-renal conditions; (6) missing data; (7) patients younger than 18 years of age. All participants or their close relatives had signed an informed consent form.

The included patients were categorized into two groups: those who developed AKI (AKI group) within seven days following a sepsis diagnosis and those who did not develop AKI (non-AKI group).

### Definitions and endpoints

The definition of sepsis 3.0 was used to define sepsis [1]. For cases before 2016, this definition was applied retrospectively. Septic shock was defined as sepsis requiring vasopressors after adequate fluid resuscitation to maintain mean arterial pressure (MAP) ≥ 65 mmHg and blood lactate concentration ≥ 2 mmol/L [1, 12]. The diagnosis and stage of AKI were based on the serum creatinine and urine output criteria proposed by Kidney Disease: Improving Global Outcomes (KDIGO) [13]. The definition of baseline creatinine was as follows: if at least five values were available, the median of all values from 6 months to 6 days before hospitalization was used; otherwise, the minimum value was five days before hospitalization [14]. If no creatinine was available before hospitalization or the emergency patient’s serum creatinine was abnormal at admission, baseline creatinine was estimated using the Modification of Diet in Renal Disease (MDRD) equation [15]. Attributable mortality of AKI was defined as the proportion of deaths that can be statistically attributed to AKI [10, 11, 16, 17]. It was calculated by subtracting the mortality of matched septic patients without AKI from the mortality of matched septic patients with AKI (i.e., attributable mortality of AKI = Mortality in matched AKI patients–Mortality in matched non-AKI patients).

The primary endpoint was 30-day mortality after sepsis diagnosis. The secondary endpoint was the ICU length of stay (LOS), hospital LOS, ICU mortality, and hospital mortality.

### Data collection

Data information included demographic characteristics [gender, age, and body mass index (BMI)], comorbidities [chronic obstructive pulmonary disease (COPD) or asthma, cardiovascular disease, hypertension, diabetes, cancer, and chronic liver disease], admission type (medical, surgical or emergency), and ICU diagnosis. The Acute Physiology and Chronic Health Evaluation II (APACHE II) score and the non-renal Sequential Organ Failure Assessment (SOFA) score were recorded on the sepsis diagnosis day. We collected clinical management data, including mechanical ventilation and renal replacement therapy (RRT), on the day of sepsis diagnosis. The mean arterial pressure (MAP) and the presence of septic shock were recorded daily within seven days of the definitive diagnosis of sepsis. We also recorded the baseline creatinine, the use of nephrotoxic drugs (angiotensin-converting enzyme inhibitors, aminoglycosides, nonsteroidal anti-inflammatory drugs, and vancomycin, et al.) [18, 19], ICU LOS, hospital LOS, ICU mortality, 30-day mortality, and hospital mortality.

### Statistical analysis

**Fig. 1:**
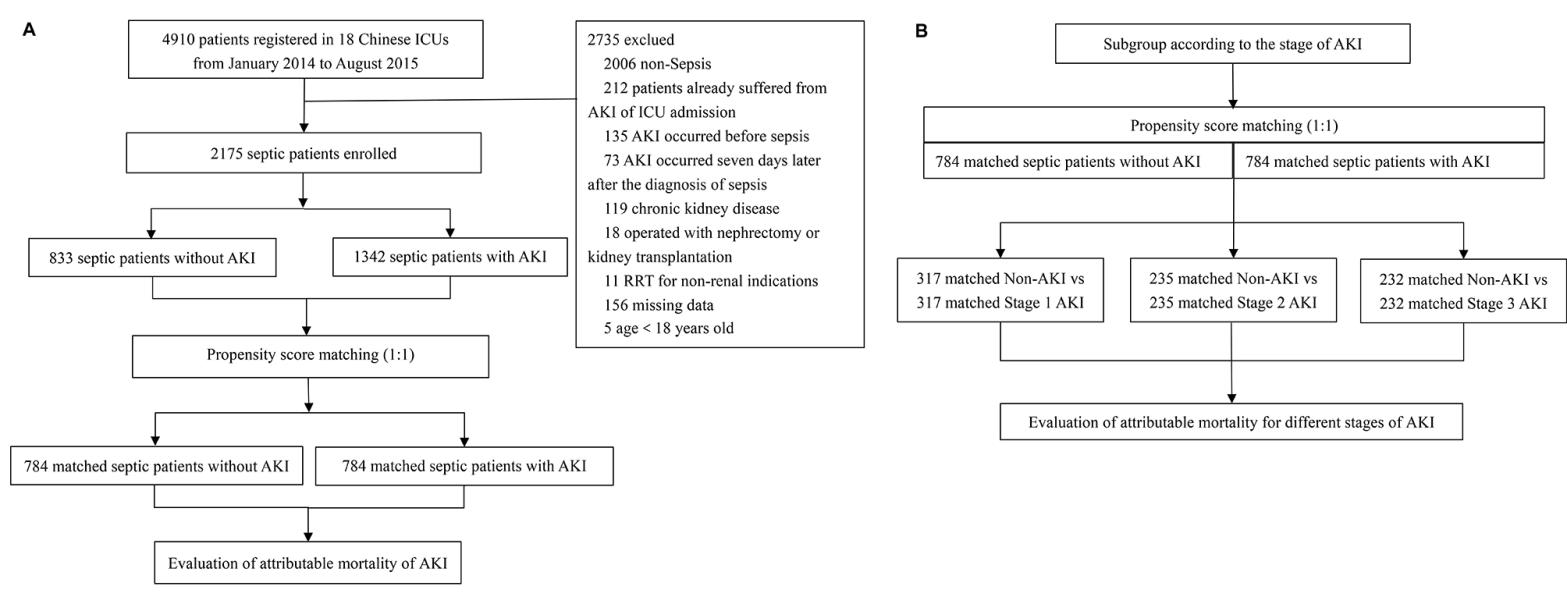
Study flow diagram (**A**) Flowchart of all the participants (**B**) Flowchart of the subgroup analysis. Abbreviations: ICU intensive care unit; AKI, acute kidney injury

Statistics were analyzed using R 4.2.3. Continuous variables were presented as medians (interquartile range, IQR). Categorical variables are expressed as percentages. For comparing continuous data, we used the Mann-Whitney U test, and for categorical variables, the Chi-square test was utilized. A significance level of *p* < 0.05 was considered to indicate statistical significance. Applying PSM between septic patients with and without AKI to estimate the excess mortality attributed to AKI (Fig. 1A). PSM was performed using the “matchit” package to balance non-renal variables on outcomes. This study used the nearest neighbor method with a caliper value of 0.06 of the standard deviation of the logit of the propensity score. The propensity score, which was based on baseline characteristics and clinical covariates, was used to adjust the differences between matched patients with and without AKI. The variables analyzed for PSM included age, gender, BMI, comorbidities, APACHE II score, non-renal SOFA score, baseline creatinine, mechanical ventilation, MAP, septic shock, and the use of nephrotoxic drugs. The variables used for propensity score matching models did not exhibit multicollinearity. The multicollinearity tests were presented in Supplemental Table S2. We checked the covariate balance using standardized differences and considered values of over 25% indicative of meaningful differences. McNemar’s test was applied to sensitivity analysis [20]. Then, the 30-day mortality in matched septic patients with and without AKI was calculated separately. Subgroup analysis of matched septic patients with and without AKI was based on the AKI stage (Fig. 1B). Attributable mortality was calculated separately for total and different stages of AKI. A 95% confidence interval (CI) for the attributable mortality difference was estimated by Newcombe’s method [21].

Survival analysis was conducted on matched AKI and matched non-AKI septic patients. We used the Kaplan-Meier survival curve to compare the survival status of both groups and the log-rank test to compare their survival time and rate differences. We also used the multivariate Cox proportional hazard model to estimate the hazard ratio (HR) and 95% confidence interval (CI) for 30-day mortality after adjusting for age, gender, BMI, comorbidities, APACHE II score, non-renal SOFA score, baseline creatinine, mechanical ventilation, RRT, MAP, septic shock, and the use of nephrotoxic drugs.

## Result

### Characteristics of the demographics and proportion of AKI

Of all 4910 patients, 2175 septic patients were included in this analysis (Table 1), which included 1342 (61.7%) cases of AKI that occurred within seven days after sepsis diagnosis (AKI group) and 833 (38.3%) cases who did not develop AKI (non-AKI group). Of the total, 1388 (63.8%) were male. The median (IQR) age was 64 (50–76) years. Patients in the AKI group had a higher BMI (*p* < 0.05). The proportion of COPD or asthma, cardiovascular disease, diabetes, cancer, and chronic liver disease was similar between the two groups. Still, the proportion of hypertension in the AKI group was higher (*p* < 0.05). 1213 (55.8%) were medical. The AKI group had higher APACHE II and non-renal SOFA scores (*p* < 0.05) and was more likely to receive mechanical ventilation and suffer from septic shock (*p* < 0.05). The septic patients in the AKI group had higher baseline creatinine and lower MAP (*p* < 0.05). In the entire cohort, the median (IQR) ICU and hospital LOS were 8 (4–17) days and 18 (10–28) days, respectively. The 30-day mortality of AKI was 31.1%.

**Table 1:** Baseline characteristics of septic patients classified by AKI

Variables	All patients *n* = 2175	Non-AKI *n* = 833	AKI *n* = 1342	*p*-value
Male gender	1388 (63.8%)	538 (64.6%)	850 (63.3%)	0.556
Age (years)	64 (50–76)	64 (49–76)	64 (50–77)	0.795
BMI (kg/m^2^)	23.3 (20.9–25.3)	22.9 (20.8–24.8)	23.4 (21.3–25.4)	< 0.001
Chronic comorbidities				
COPD/asthma	167 (7.7%)	64 (7.7%)	103 (7.7%)	0.995
Cardiovascular disease	320 (14.7%)	108 (13.0%)	212 (15.8%)	0.070
Hypertension	687 (31.6%)	238 (28.6%)	449 (33.5%)	0.017
Diabetes	397 (18.3%)	147 (17.6%)	250 (18.6%)	0.564
Cancer	224 (10.3%)	86 (10.3%)	138 (10.3%)	0.976
Chronic liver disease	40 (1.8%)	11 (1.3%)	29 (2.2%)	0.156
Admission type				
Medical	1213 (55.8%)	441 (52.9%)	772 (57.5%)	0.051
Surgical	470 (21.6%)	201 (24.1%)	269 (20.0%)	
Emergency	492 (22.6%)	191 (22.9%)	301 (22.4%)	
APACHE II score	19 (14–25)	18 (13–23)	20 (14–26)	< 0.001
Non-renal SOFA score	7 (4–10)	6 (4–9)	8 (5–11)	< 0.001
Mechanical ventilation	1707 (78.5%)	626 (75.2%)	1081 (80.6%)	0.003
Baseline creatinine	77 (66–88)	75 (62–86)	79 (69–89)	< 0.001
Use of nephrotoxic drugs	166 (7.6%)	64 (7.7%)	102 (7.6%)	0.944
Septic shock	1067 (49.1%)	312 (37.5%)	755 (56.3%)	< 0.001
MAP (mmHg)	75 (60–90)	79 (66–94)	72 (60–87)	< 0.001
Outcomes				
ICU LOS (days)	8 (4–17)	7 (3–16)	9 (5–17)	< 0.001
Hospital LOS (days)	18 (10–28)	18 (10–28)	18 (10–28)	0.679
ICU mortality	337 (15.5%)	78 (9.4%)	259 (19.3%)	< 0.001
30-day mortality	590 (27.1%)	173 (20.8%)	417 (31.1%)	< 0.001
Hospital mortality	788 (36.2%)	244 (29.3%)	544 (40.5%)	< 0.001

Abbreviations: AKI, acute kidney injury; BMI, body mass index; COPD, chronic obstructive pulmonary disease; APACHE II, acute physiologic and chronic health evaluation II; SOFA, sequential organ failure assessment; MAP, mean arterial pressure; LOS, length of stay; ICU, intensive care unit

Among 1342 AKI patients, 474 (35.3%) were stage 1, 401 (29.9%) were stage 2, and 467 (34.8%) were stage 3. The 30-day mortality was 23.0%, 27.7%, and 42.2%, respectively (*p* < 0.05). The baseline characteristics of different stages of AKI were shown in supplemental material Table S1.

### Attributable mortality of AKI in septic patients

**Fig. 2:**
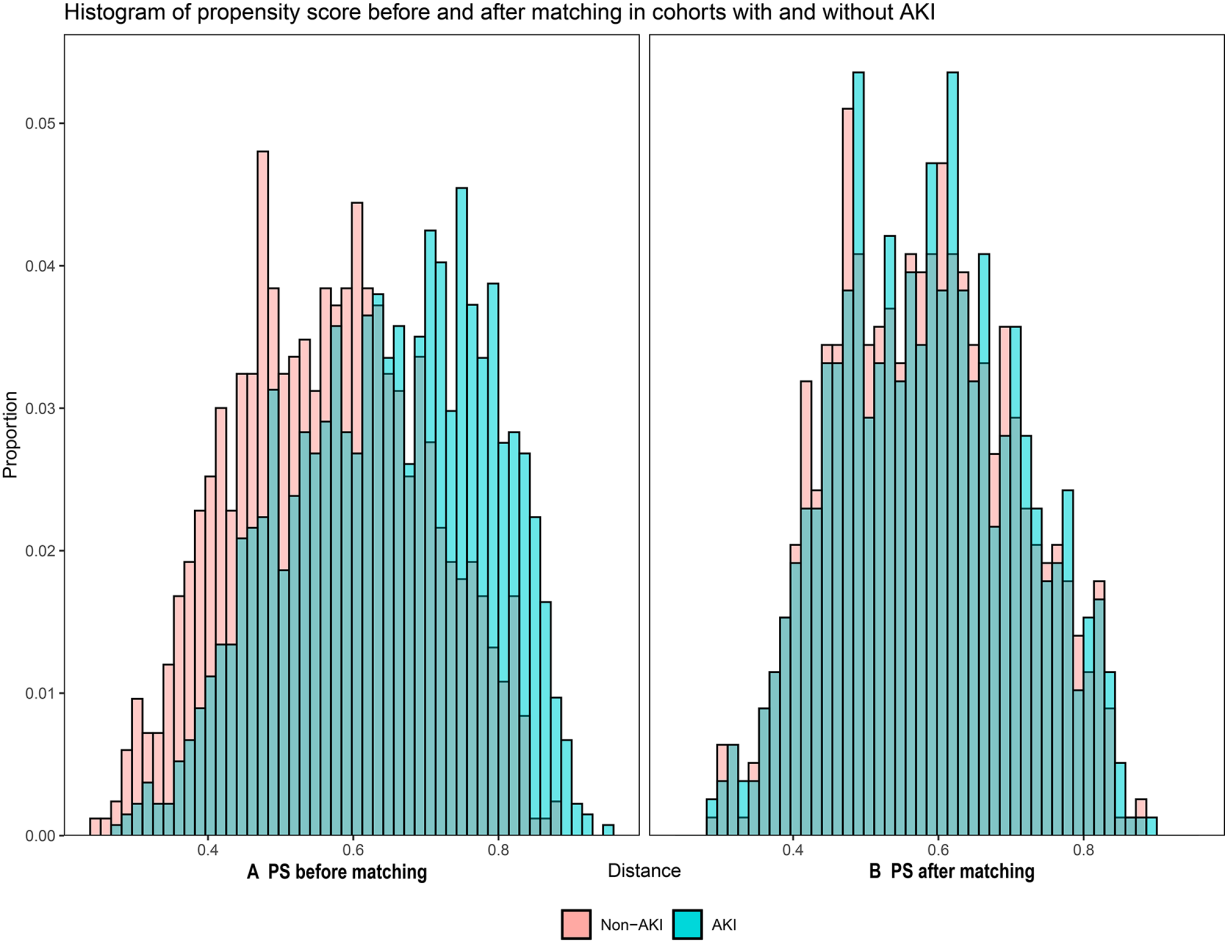
Histograms of propensity score before and after matching in cohorts with and without AKI (**A**) PS before matching (**B**) PS after matching. Abbreviations: PS, propensity score

Of the 2175 septic patients, 784 patients with AKI were matched 1:1 with 784 non-AKI patients, according to PSM. Characteristics and standardized differences of the matched patients in the two groups were shown in Table 2. After PSM, no variable exhibited a significant imbalance. The histograms of propensity score before and after matching in cohorts with and without AKI were shown in Fig. 2. The density plot of propensity score before and after matching in cohorts with and without AKI was presented in Supplemental material Fig. S1. The Q-Q plots of the balance of the covariates were shown in Supplemental material Fig. S2. The Jitter plot of the distribution of propensity scores was shown in Supplemental material Fig. S3. The standardized difference before and after matching in cohorts with and without AKI was shown in Supplemental material Fig. S4.

**Table 2:** Characteristics between matched septic patients with and without AKI

Variables	Non-AKI *n* = 784	AKI *n* = 784	*p*- value	Standardized difference(%)
Male gender	504 (64.3%)	504 (64.3%)	1.000	10.5
Age (years)	64 (49–76)	64 (49–77)	0.578	6.5
BMI (kg/m^2^)	23.1 (20.8–24.9)	23.0 (20.8–25.1)	0.950	13.5
Chronic comorbidities				
COPD/asthma	62 (7.9%)	62 (7.9%)	1.000	8.3
Cardiovascular disease	107 (13.7%)	107 (13.7%)	1.000	8.1
Hypertension	230 (29.3%)	226 (28.8%)	0.824	1.6
Diabetes	138 (17.6%)	138 (17.6%)	1.000	1.0
Cancer	77 (9.8%)	76 (9.7%)	0.932	0.3
Chronic liver disease	11 (1.4%)	14 (1.8%)	0.545	19.2
APACHE II score	18 (13–23)	18 (13–23)	0.654	16.6
Non-renal SOFA score	6 (4–9)	6 (4–9)	0.473	18.7
Baseline creatinine	76 (64–87)	76 (65–88)	0.539	9.4
Mechanical ventilation	592 (75.5%)	611 (77.9%)	0.256	17.5
Use of nephrotoxic drugs	61 (7.8%)	70 (8.9%)	0.411	4.6
Septic shock	311 (39.7%)	333 (42.5%)	0.259	5.5
MAP (mmHg)	78 (65–93)	77 (64–92)	0.565	13.8

Abbreviations: AKI, acute kidney injury; BMI, body mass index; COPD, chronic obstructive pulmonary disease; APACHE II, acute physiologic and chronic health evaluation II; SOFA, sequential organ failure assessment; MAP, mean arterial pressure

**Fig. 3:**
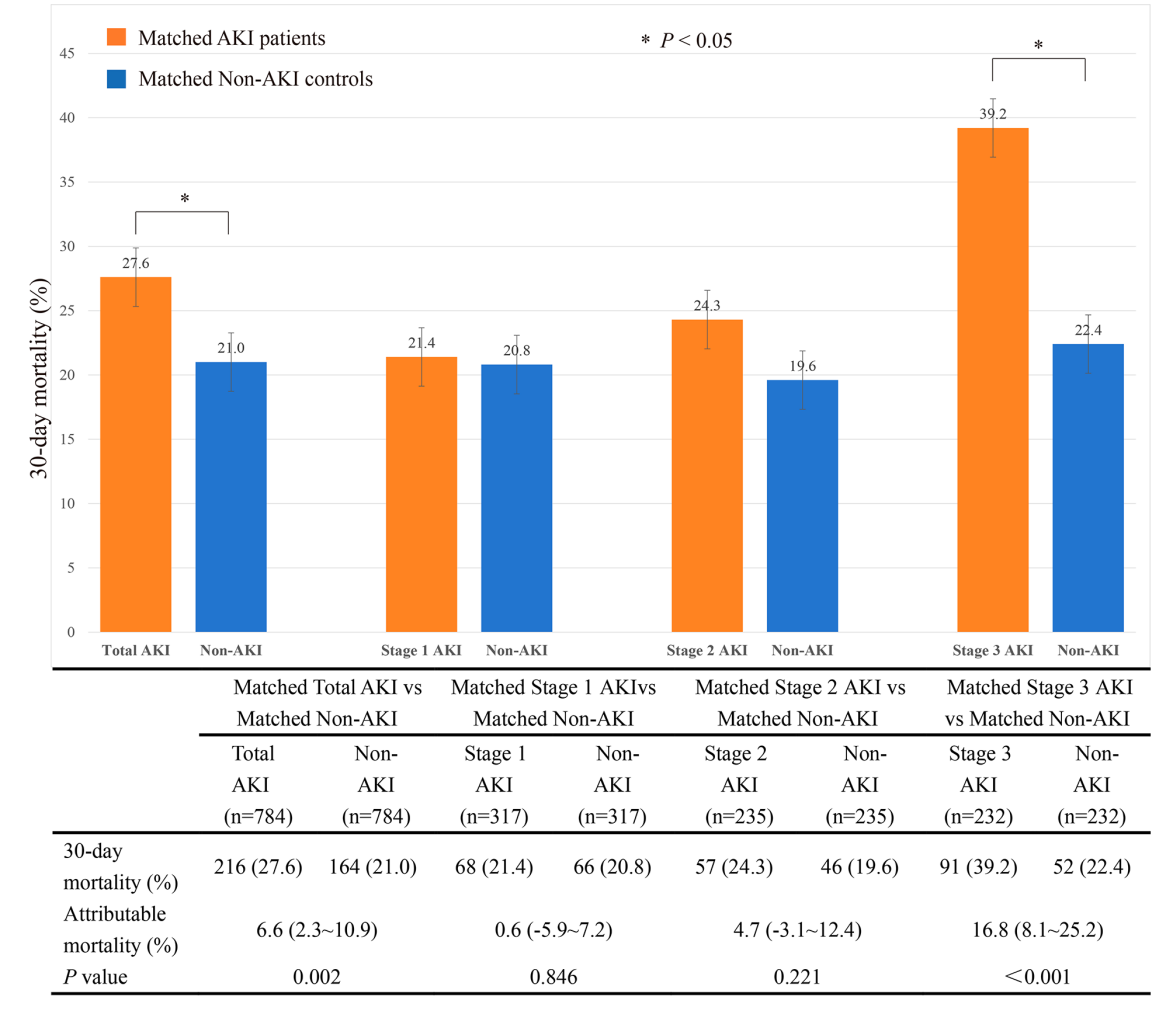
The attributable mortality of total AKI and different stages of AKI in subgroups Abbreviations: AKI, acute kidney injury

The 30-day mortality of the matched septic patients with total AKI was 216 of 784 (27.6%) compared with 164 of 784 (21.0%) for septic patients without AKI (*p* < 0.001). The 30-day attributable mortality of total AKI was 6.6% (95% CI 2.3 ∼ 10.9%, *p* = 0.002). Matched septic patients with AKI were subgrouped according to the AKI stage. A total of 317 patients with stage 1 AKI, 235 patients with stage 2 AKI, and 232 patients with stage 3 AKI were matched 1:1 with their controls without AKI, respectively. Subgroup analysis showed the 30-day attributable mortality of stage 1, stage 2, and stage 3 AKI was 0.6% (95% CI −5.9 ∼ 7.2%, *p* = 0.846), 4.7% (95% CI −3.1 ∼ 12.4%, *p* = 0.221) and 16.8% (95% CI 8.1 ∼ 25.2%, *p* < 0.001), respectively, as shown in Fig. 3.

### Sensitivity analysis

A sensitivity analysis was conducted to assess the impact of unmeasured covariates on the stability of results. The gamma parameter was used to measure the stability of results, which reflected the probability of differential assignment to treatment due to unobserved factors [22, 23]. The effect estimate was considered robust to hidden bias if the statistical significance did not change until a large gamma. The results of this study showed that the estimation of 30-day attributable mortality of AKI was reliable for the hidden bias of unmeasured covariates, with a gamma coefficient of up to 3. The sensitivity analysis of 30-day attributable mortality results was shown in supplemental material Table S3.

### Survival analysis

**Fig. 4:**
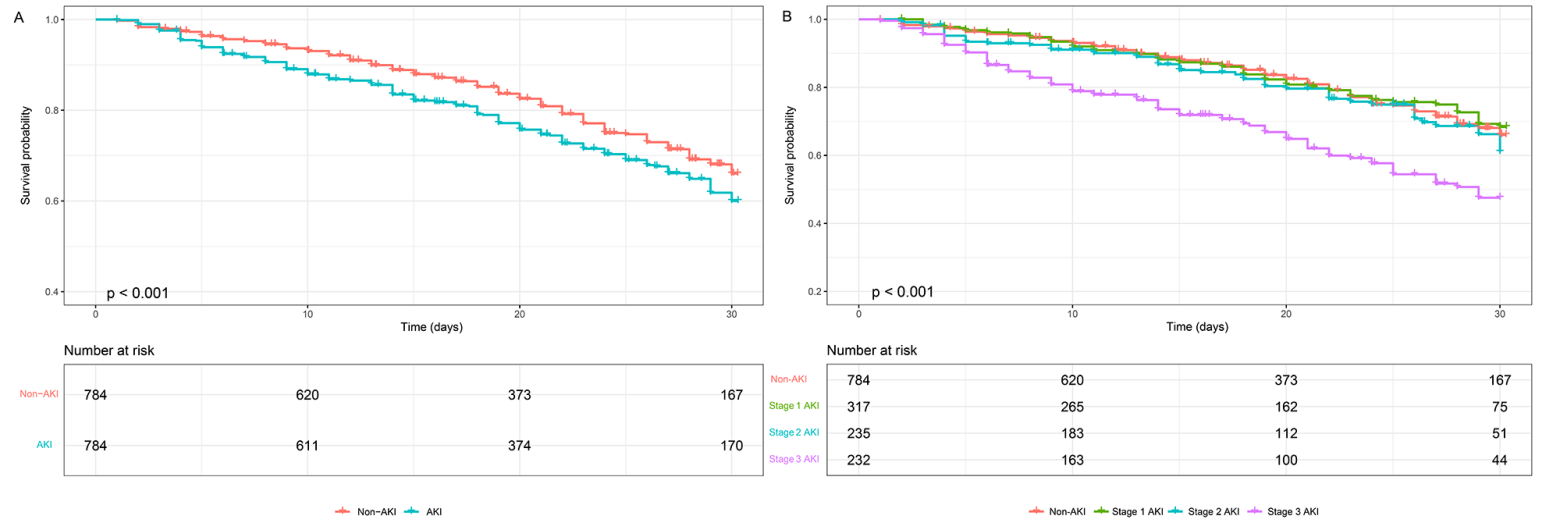
Kaplan-Meier survival analysis (**A**) Kaplan-Meier survival analysis comparing non-AKI and AKI groups (**B**) Kaplan-Meier survival analysis comparing non-AKI, stage 1 AKI, stage 2 AKI, and stage 3 AKI groups. *p* < 0.001 for all comparisons. The numbers of patients at risk at each time point were shown below the graph. Abbreviations: AKI, acute kidney injury

In Fig. 4A, the Kaplan-Meier curve revealed significantly higher 30-day mortality in the AKI group compared to the non-AKI group (*p* < 0.001). Survival probabilities at the 30-day were 66.0% (95% CI 61.5 ∼ 70.8%) for non-AKI patients and 60.0% (95% CI 55.6 ∼ 64.8%) for AKI patients. Subgroup analysis demonstrated statistical significance between stage 1 and stage 3 AKI, as well as stage 2 and stage 3 AKI (*p* < 0.001), as shown in Fig. 4B. Survival probabilities at the 30-day were 68.4% (95% CI 61.9 ∼ 75.5%) for stage 1 AKI patients, 61.0% (95% CI 52.7 ∼ 70.7%) for stage 2 AKI patients, and 47.6% (95% CI 40.0 ∼ 56.5%) for stage 3 AKI patients.

**Fig. 5:**
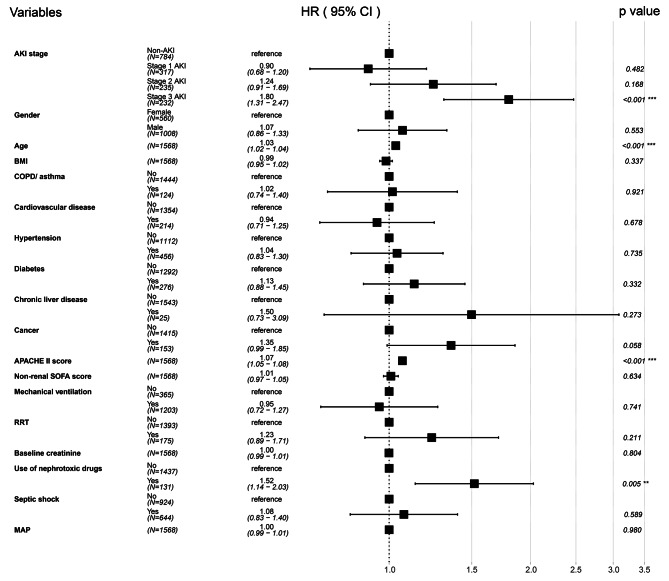
Forest plot of multivariable Cox proportional hazard regression analysis for 30-day mortality stratified by AKI stage. Abbreviations: AKI, acute kidney injury; BMI, body mass index; COPD, chronic obstructive pulmonary disease; APACHE II, acute physiologic and chronic health evaluation II; SOFA, sequential organ failure assessment; RRT, renal replacement therapy; MAP, mean arterial pressure

In our multivariate Cox regression adjusted model (Fig. 5), stage 3 AKI was associated with higher 30-day mortality, with an adjusted HR of 1.80 (95% CI 1.31 ∼ 2.47, *p* < 0.001). However, stage 1 and stage 2 AKI did not increase 30-day mortality, with adjusted HR of 0.90 (95% CI 0.68 ∼ 1.20, *p* = 0.482) and 1.24 (95% CI 0.91 ∼ 1.69, *p* = 0.168), respectively. Supplemental material Table S4 showed the multivariable Cox proportional hazard regression analysis for 30-day mortality stratified by AKI stage.

## Discussion

Sepsis is the leading cause of AKI [24]. Sepsis-related AKI is a common complication among critically ill patients and is associated with high morbidity and mortality [25]. One study that enrolled critically ill patients from 24 European countries reported an incidence rate of AKI in septic patients of 51%, and the mortality in ICU was 41% [8]. In contrast, another study that enrolled hospitalized patients across China reported a rate of 47.1% [9]. Recent evidence suggests that microvascular dysfunction, inflammation, and metabolic reprogramming may be the mechanisms leading to AKI in sepsis [7]. Since mortality rates are significantly influenced by patient demographics and the varying levels of care provided to critically ill patients with sepsis, the precise excess mortality attributed to AKI may differ considerably across different healthcare facilities. Calculating the attributable mortality of AKI would provide insight into the number of preventable deaths if AKI could be avoided [10]. We reported the proportion of deaths attributable to AKI in septic patients. This finding would help to calculate the sample size for future clinical trials of AKI in ICU septic patients. Our research found that the overall 30-day attributable mortality of AKI in septic patients was 6.6%. Subgroup analysis showed that the excess 30-day mortality of stage 3 AKI was 16.8%; stage 1 and stage 2 AKI did not increase the excess 30-day mortality, highlighting the importance of early recognition and treatment of AKI in septic patients. More aggressive treatment could help prevent the progression of AKI and improve the chances of survival, such as renal replacement therapy or immunomodulatory agents.

In 2014, Vaara ST conducted a study on 954 patients in a general ICU, which found that AKI lead to a 90-day absolute excess mortality rate of 8.6%. The study’s subgroup analysis revealed that patients in stage 1 AKI had a 90-day attributable mortality rate of 1.4%, while it was 12.0% for stage 2 AKI patients and a staggering 16.7% for stage 3 AKI patients [10]. A study performed by Jiang Yijia demonstrated that the attributable mortality for new-onset AKI in ICU patients was 8.1%. Interestingly, stage 1 AKI did not increase mortality, but stage 2 and stage 3 AKI had attributable mortality rates of 17.1% and 18.2%, respectively [11]. Our study discovered that stage 2 AKI did not increase the 30-day mortality rate of septic patients. This differed from the two similar studies, which included all general ICU patients. However, our study only focused on septic patients, which may account for the disparity in results. According to Wang Zhiyi’s research [26], the Medical Information Mart for Intensive Care (MIMIC) IV cohort, consisting of 15,610 patients, had an excess attributable mortality rate of 58.6% for AKI in critically ill patients with sepsis. In contrast, in the Wenzhou cohort, consisting of 1,341 patients, the excess attributable mortality was 44.6%. Wang Zhiyi’s results were higher than ours, probably because of the different approach used than the present study. They applied a directed acyclic graph (DAG) approach, which eliminated potential confounders and mediating variables, to calculate the attributable mortality rate for AKI. These studies all indicated that septic patients with stage 3 AKI had a poor prognosis. However, in a study meticulously conducted by Fujii in Japan, encompassing 424 septic patients and utilizing the sepsis 3.0 definition and KDIGO criteria, the anticipated impact of AKI on hospital mortality among the septic cohort remained elusive [27]. In contrast, our investigation revealed that stage 3 AKI notably elevated the 30-day mortality rate among septic patients. This divergence from the Japanese study could potentially be attributed to the relatively smaller sample size of septic patients, possibly obscuring any significant differences that may have otherwise been observed. The studies on the attributable mortality of AKI vary significantly among different studies, possibly due to the differences in race, healthcare resources availability, or clinical practice.

Our research demonstrated that the survival rate of stage 3 AKI was significantly lower, at only 47.6%. Cheyron’s study found that only severe AKI showed a significant association with excess attributable mortality in ICU patients diagnosed with liver cirrhosis [28]. Jiang YiJia reported in another study that new-onset AKI was a significant risk factor for 28-day mortality and persistent AKI was strongly linked to unfavorable outcomes [29]. Furthermore, our Cox regression analysis found that stage 3 AKI was an independent risk factor for 30-day mortality, with a mortality rate 0.80 times higher than that of non-AKI patients in the ICU. These findings suggested that early intervention and targeted treatment strategies may be critical in septic patients with stage 1–2 AKI, preventing kidney function deterioration from progressing to stage 3 AKI.

There were several highlights in this study. The data came from a large, multicenter, prospective clinical study in China, strengthening the results’ validity and generality. Secondly, we applied the definition of sepsis 3.0 and the KDIGO criteria for AKI. Thirdly, from a clinical perspective, our findings emphasize the critical need for healthcare professionals to remain vigilant when septic patients develop AKI. Preventing the progression to stage 3 AKI is imperative to ensure the best possible patient outcomes. However, there were also a few drawbacks in this study. It was imperative to acknowledge that the study under review was a retrospective secondary analysis, which may introduce unmeasured bias. The study engaged only those septic patients admitted to the ICU within seven days. However, additional research is required to comprehensively address cases where sepsis develops beyond this period. Consequently, additional observational studies are needed to gain a deeper and more comprehensive understanding of the findings.

## Conclusion

The overall 30-day attributable mortality of AKI among critically ill patients with sepsis was 6.6%. Stage 3 AKI had the most significant contribution to 30-day mortality, while stage 1 and stage 2 AKI did not increase excess mortality. Stage 3 AKI was an independent risk factor for 30-day mortality.

### Electronic supplementary material

Below is the link to the electronic supplementary material.

Supplementary Material 1

## Data Availability

The datasets analyzed during the current study are available from the corresponding author upon reasonable request.

## Abbreviations

AKI: Acute kidney injury
ICU: Intensive care unit
PSM: Propensity score matching
BMI: Body mass index
COPD: Chronic obstructive pulmonary disease
CKD: Chronic kidney disease
APACHE II: Acute physiologic and chronic health evaluation II
SOFA: Sequential organ failure assessment
RRT: Renal replacement therapy
MAP: Mean arterial pressure
LOS: Length of stay
IQR: Interquartile range
HR: Hazard ratio
CI: Confidence interval
