# Supplementary material for: Attributable mortality of acute kidney injury among critically ill patients with sepsis: a multicenter, retrospective cohort study
Source: BMC Nephrol. 2024 Apr 8;25:125. doi: 10.1186/s12882-024-03551-9 (PMC11000341; doi:10.1186/s12882-024-03551-9)
Supplement: Supplementary file 1 — Supplementary Material 1 [file 12882_2024_3551_MOESM1_ESM.docx]

**Attributable mortality of acute kidney injury among critically ill patients with sepsis: a multicenter, retrospective cohort study**

**Supplemental Content**

**Dong-Hui Wang^1^**^†^**, Jin-Chao Zhao^2^**^†^, **Xiu-Ming Xi^3^, Yue Zheng^1*^, Wen-Xiong Li^1*^**

^1^Department of Surgical Intensive Care Unit, Beijing Chao-yang Hospital, Capital Medical University, Beijing, China

^2^Department of Clinical Laboratory, Xiangyang No.1 People’ s Hospital, Hubei University of Medicine, Xiangyang, China

^3^Department of Critical Care Medicine, Fuxing Hospital, Capital Medical University, Beijing, China

^†^Dong-Hui Wang and Jin-Chao Zhao contributed equally to this work.

**Table of Contents (in order of appearance in manuscript)**:

Supplemental Table S1 Baseline characteristics of different stages of AKI

Supplemental Table S2 Multicollinearity tests of variables used for propensity score matching models

Supplemental Fig. S1 Density plot of propensity score before and after matching in cohorts with and without AKI

Supplemental Fig. S2 The Q-Q plots of the balance of the covariates

Supplemental Fig. S3 The Jitter plots of the distribution of propensity scores

Supplemental Fig. S4 The standardized difference before and after matching in cohorts with and without AKI

Supplemental Table S3 Results of the sensitivity analysis of 30-day attributable mortality

Supplemental Table S4 Multivariable Cox proportional hazard regression analysis for 30-day mortality stratified by AKI stage

**Supplemental Table S1**

Baseline characteristics of different stages of AKI

| **Variables** | **Stage 1 AKI**  **n=474** | **Stage 2 AKI**  **n=401** | **Stage 3 AKI**  **n=467** | ***p* value** |
| --- | --- | --- | --- | --- |
| Male gender | 324 (68.4%) | 234 (58.4%) | 292 (62.5%) | 0.008 |
| Age (years) | 66 (51-78) | 62 (49-76) | 64 (48-76) | 0.046 |
| BMI (kg/m^2^) | 23.2 (21.3-25.2) | 23.3 (20.8-25.3) | 23.7 (21.4-25.7) | 0.060 |
| Chronic comorbidities | | | | |
| COPD/asthma | 52 (11.0%) | 30 (7.5%) | 21 (4.5%) | < 0.001 |
| Cardiovascular disease | 87 (18.4%) | 62 (15.5%) | 63 (13.5%) | 0.117 |
| Hypertension | 156 (32.9%) | 131 (32.7%) | 162 (34.7%) | 0.780 |
| Diabetes | 86 (18.1%) | 79 (19.7%) | 85 (18.2%) | 0.809 |
| Cancer | 45 (9.5%) | 32 (8.0%) | 61 (13.1%) | 0.035 |
| Chronic liver disease | 11 (2.3%) | 8 (2.0%) | 10 (2.1%) | 0.970 |
| Admission type | | | | |
| Medical | 231 (48.7%) | 226 (56.4%) | 315 (67.5%) | < 0.001 |
| Surgical | 127 (26.8%) | 94 (23.4%) | 48 (10.3%) | < 0.001 |
| Emergency | 116 (24.5%) | 81 (20.2%) | 104 (22.3%) | < 0.001 |
| APACHE II score | 18 (13-24) | 18 (14-24) | 23 (17-28) | < 0.001 |
| Non-renal SOFA score | 7 (4-10) | 8 (5-11) | 10 (7-14) | < 0.001 |
| Mechanical ventilation | 381 (80.4%) | 324 (80.8%) | 376 (80.5%) | 0.994 |
| RRT | 22 (4.6%) | 42 (10.5%) | 268 (57.4%) | < 0.001 |
| Baseline creatinine | 77 (67-88) | 80 (70-89) | 79 (69-89) | 0.077 |
| Use of nephrotoxic drugs | 43 (9.1%) | 28 (7.0%) | 31 (6.6%) | 0.329 |
| Septic shock | 229 (48.3%) | 214 (53.4%) | 312 (66.8%) | < 0.001 |
| MAP (mmHg) | 77 (62-92) | 73 (60-86) | 64 (58-80) | < 0.001 |
| Outcomes | | | | |
| ICU LOS (days) | 9 (5-17) | 9 (5-17) | 9 (5-17) | 0.907 |
| Hospital LOS (days) | 20 (13-29) | 18 (10-28) | 15 (8-26) | < 0.001 |
| ICU mortality | 55 (11.6%) | 75 (18.7%) | 129 (27.6%) | < 0.001 |
| 30-day mortality | 109 (23.0%) | 111 (27.7%) | 197 (42.2%) | < 0.001 |
| Hospital mortality | 176 (37.1%) | 129 (32.2%) | 239 (51.2%) | < 0.001 |

Abbreviations: AKI, acute kidney injury; BMI, body mass index; COPD, chronic obstructive pulmonary disease; APACHE II, acute physiologic and chronic health evaluation II; SOFA, sequential organ failure assessment; RRT, renal replacement therapy; MAP, mean arterial pressure; LOS, length of stay; ICU, intensive care unit

**Supplemental Table S2**

Multicollinearity tests of variables used for propensity score matching models

| **Variables** | **Tolerance** | **Variance Inflation Factor(VIF)** |
| --- | --- | --- |
| gender | 0.969 | 1.032 |
| age | 0.736 | 1.359 |
| BMI | 0.961 | 1.040 |
| COPD/asthma | 0.933 | 1.071 |
| Cardiovascular disease | 0.829 | 1.206 |
| Hypertension | 0.763 | 1.310 |
| Diabetes | 0.862 | 1.161 |
| Cancer | 0.959 | 1.042 |
| Chronic liver disease | 0.987 | 1.013 |
| APACHE II score | 0.634 | 1.578 |
| Non-renal SOFA score | 0.533 | 1.876 |
| Baseline creatinine | 0.944 | 1.059 |
| Mechanical ventilation | 0.931 | 1.074 |
| Use of nephrotoxic drugs | 0.978 | 1.023 |
| Septic shock | 0.574 | 1.743 |
| MAP | 0.663 | 1.508 |


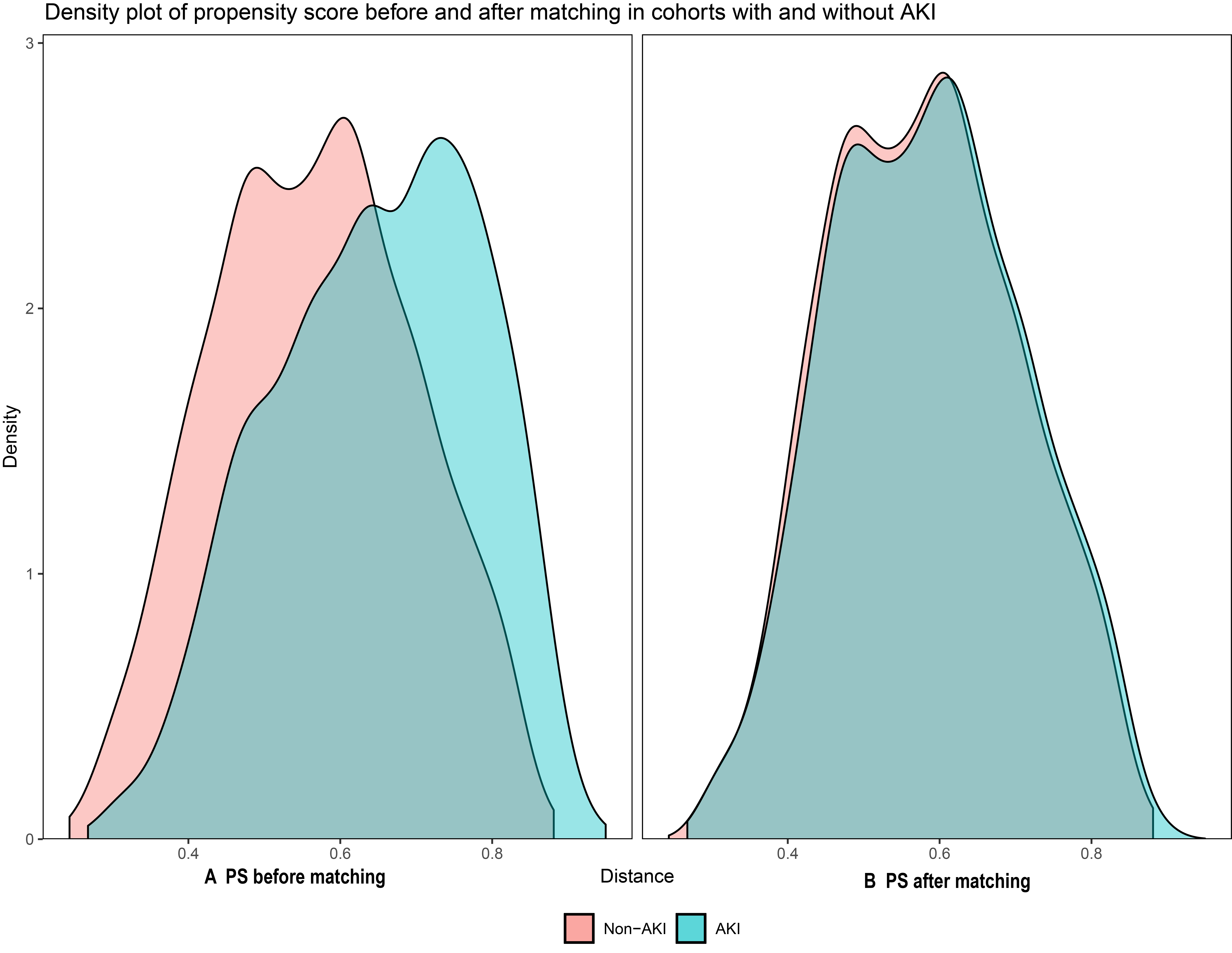


**Supplemental Fig. S1**

Density plot of propensity score before and after matching in cohorts with and without AKI. (A) PS before matching. (B) PS after matching. Abbreviations: PS, propensity score





**Supplemental Fig. S2**

The Q-Q plots of the balance of the covariates

Abbreviations: AKI, acute kidney injury; BMI, body mass index; COPD, chronic obstructive pulmonary disease; APACHE II, acute physiologic and chronic health evaluation II; SOFA, sequential organ failure assessment; RRT, renal replacement therapy; MAP, mean arterial pressure; ICU, intensive care unit


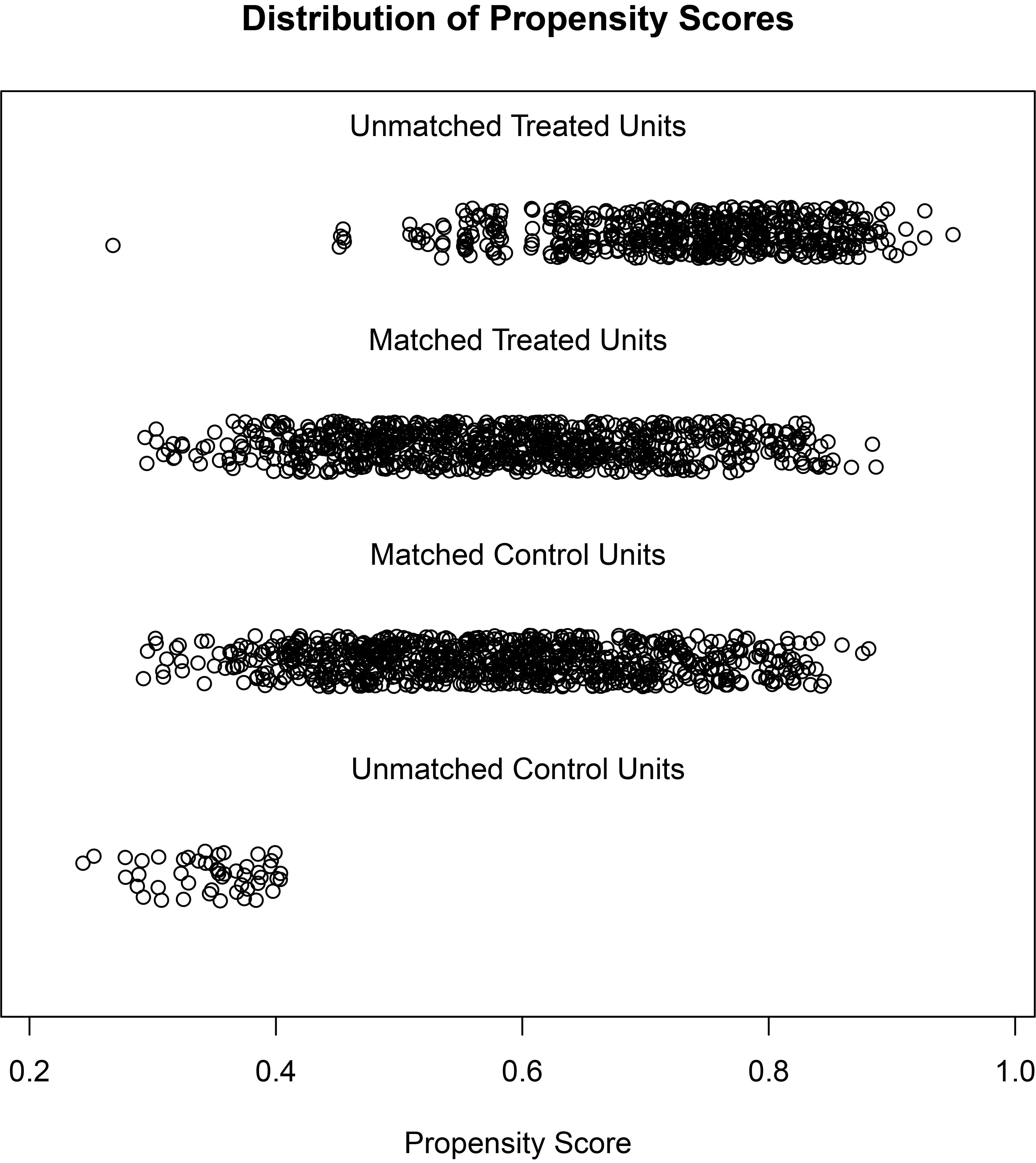


**Supplemental Figure S3**

The Jitter plots of the distribution of propensity scores


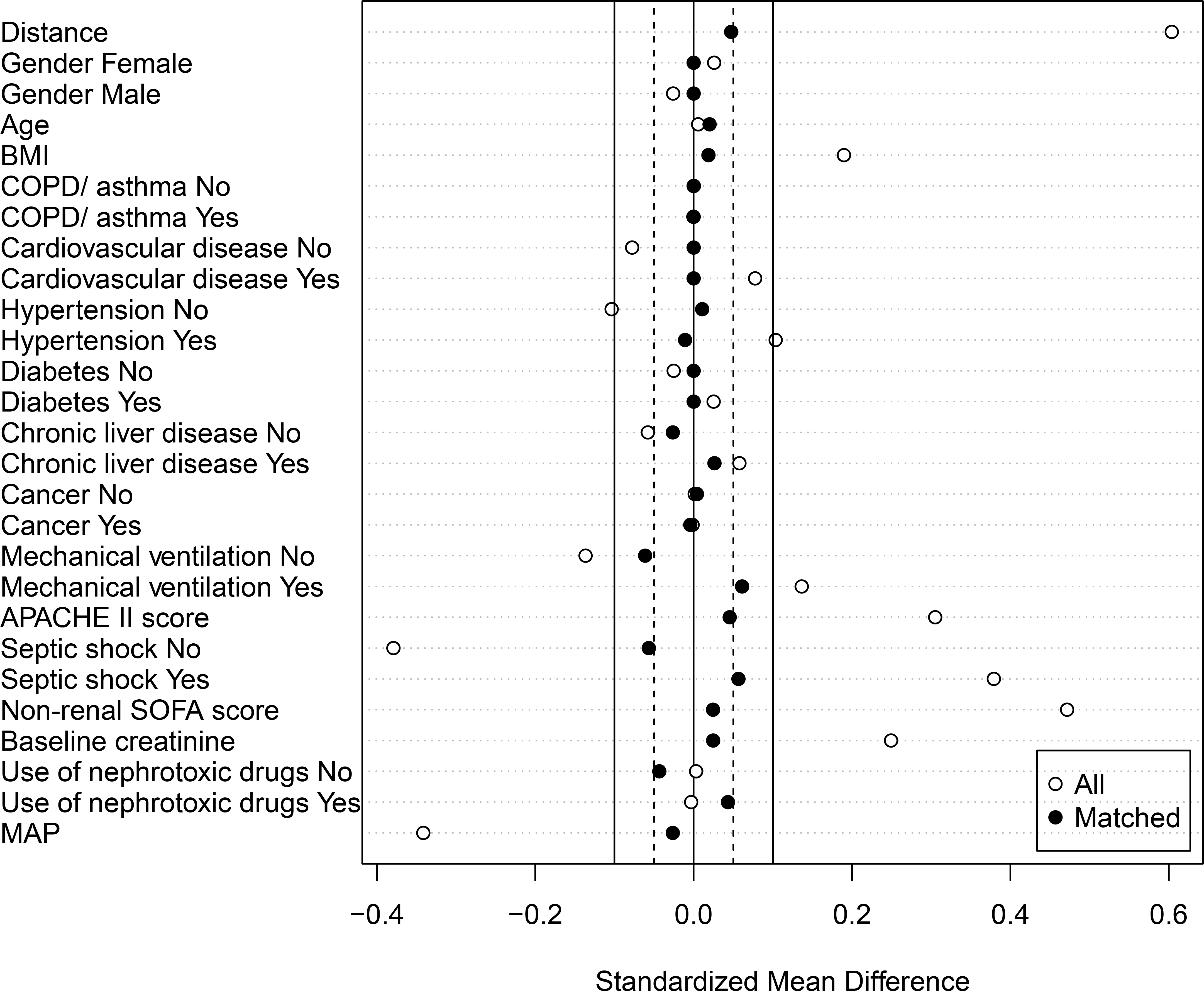


**Supplemental Fig. S4**

The standardized difference before and after matching in cohorts with and without AKI

Abbreviations: AKI, acute kidney injury; BMI, body mass index; COPD, chronic obstructive pulmonary disease; APACHE II, acute physiologic and chronic health evaluation II; SOFA, sequential organ failure assessment; MAP, mean arterial pressure.

**Supplemental Table S3**

Results of the sensitivity analysis of 30-day attributable mortality

| **Gamma** | **Lower bound** | **Upper bound** |
| --- | --- | --- |
| 1 | 0 | 0.00000 |
| 2 | 0 | 0.00000 |
| 3 | 0 | 0.04668 |
| 4 | 0 | 0.94146 |
| 5 | 0 | 0.99996 |
| 6 | 0 | 1.00000 |

**Supplemental Table S4**

Multivariable Cox proportional hazard regression analysis for 30-day mortality stratified by AKI stage

| **Variables** | **HR (95% CI)** | ***p-*value** |
| --- | --- | --- |
| AKI Stage | | |
| Stage 1 AKI | 0.90 (0.68-1.20) | 0.482 |
| Stage 2 AKI | 1.24 (0.91-1.69) | 0.168 |
| Stage 3 AKI | 1.80 (1.31-2.47) | < 0.001 |
| Male gender | 1.07 (0.86-1.33) | 0.553 |
| Age | 1.03 (1.02-1.04) | < 0.001 |
| BMI | 0.99 (0.95-1.02) | 0.337 |
| Chronic comorbidities | | |
| COPD/asthma | 1.02 (0.74-1.40) | 0.921 |
| Cardiovascular disease | 0.94 (0.71-1.25) | 0.678 |
| Hypertension | 1.04 (0.83-1.30) | 0.735 |
| Diabetes | 1.13 (0.88-1.45) | 0.332 |
| Chronic liver disease | 1.50 (0.73-3.09) | 0.273 |
| Cancer | 1.35 (0.99-1.85) | 0.058 |
| APACHE II score | 1.07 (1.05-1.08) | < 0.001 |
| Non-renal SOFA score | 1.01 (0.97-1.05) | 0.634 |
| Mechanical ventilation | 0.95 (0.72-1.27) | 0.741 |
| RRT | 1.23 (0.89-1.71) | 0.211 |
| Baseline creatinine | 1.00 (0.99-1.01) | 0.804 |
| Use of nephrotoxic drugs | 1.52 (1.14-2.03) | 0.005 |
| Septic shock | 1.08 (0.83-1.40) | 0.589 |
| MAP (mmHg) | 1.00 (0.99-1.01) | 0.980 |

Abbreviations: AKI, acute kidney injury; BMI, body mass index; COPD, chronic obstructive pulmonary disease; APACHE II, acute physiologic and chronic health evaluation II; SOFA, sequential organ failure assessment; RRT, renal replacement therapy; MAP, mean arterial pressure
